# Supplementary material for: Cystic Fibrosis Defective Response to Infection Involves Autophagy and Lipid Metabolism
Source: Cells. 2020 Aug 6;9(8):1845. doi: 10.3390/cells9081845 (PMC7463682; doi:10.3390/cells9081845)
Supplement: Supplementary file 1 [file cells-09-01845-s001.zip › Mingione_et_al_Supplementary_Figure 1.pdf]

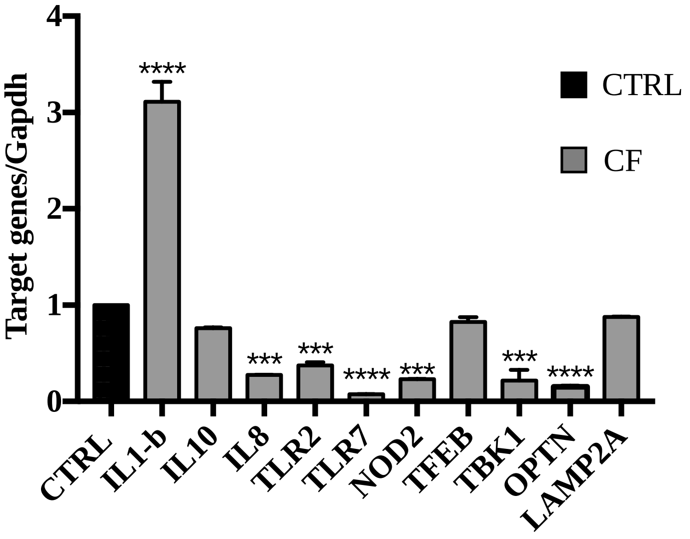

Supplementary figure 1. Quantification of the expression of genes involved in inflammation, response to infection and autophagy: pro-inflammatory *IL1β* interleukin; pro-inflammatory *IL-8* chemokine; anti-inflammatory *IL10* interleukin; Pathogens recognition receptors (PRRs): *NOD2*, *TLR2* and *TLR7*; *TFEB*; *TBK1*; *OPTN*; *LAMP2a* by qRT-PCR in CF versus CTR bronchial epithelial cells. *GAPDH* was used as a housekeeping gene. Data, derived for triplicate samples, are expressed as mean±SE (*** $p<0.001$; **** $p<0.0001$); 2-way Anova followed by Bonferroni correction was used for all data.
