# Supplementary material for: Cystic Fibrosis Defective Response to Infection Involves Autophagy and Lipid Metabolism
Source: Cells. 2020 Aug 6;9(8):1845. doi: 10.3390/cells9081845 (PMC7463682; doi:10.3390/cells9081845)
Supplement: Supplementary file 1 [file cells-09-01845-s001.zip › Mingione_et_al_Supplementary_Table1.pdf]

Supplementary Table 1: demographics and clinical features of cohort 1 (F508del/F508del) and cohort 2 (F508del/other)

	Cohort 1 (n=22)	Cohort 2 (n=21)
Genotype (%)	F508del/F508del (100)	F508del/N1303K (33,3)<br>F508del/G542X (28,6)<br>F508del/G85E (19,0)<br>F508del/del es 22-24 (14,3)<br>F508del/W1282X (4,8)
Gender (F/M)	9/13	15/6
Average age (years)	22,4	29,6
Sweat chloride > 60mmol/L	22 (100)	21 (100)
Median FEV1 (% pred); range	65; 22-118	72; 39-98
Median BMI; range	19,48; 15,20-23,46	20,66; 14,95-23,53
Clinical features:		
- Pancreas insufficiency (%)	22 (100)	19 (90,5)
- CFRD (%)	3 (13,6)	6 (28,6)
- Liver disease		6 (28,6)
- Nasal polyps (%)	9 (40,1)	5 (23,8)
- Malnutrition (%)	3 (13,6)	3 (14,2)
- Respiratory failure	4 (18,1)	1 (4,8)
- >2 pulmonary exacerbations in the last 12 months (%)	9 (40,1)	12 (57,1)
- Lung transplant (%)	0 (0)	1 (4,8)
N° patients on CFTR modulators therapy	12 (54,5)	0 (0)
Chronic lung infection		
- Pseudomonas aeruginosa	13 (59,1)	15 (71,4)
- MSSA	18 (81,8)	19 (90,4)
- MRSA	1 (4,5)	2 (9,5)
- Aspergillus fumigatus_	4 (18,1)	8 (38,1)

CFRD = CF related diabetes; MSSA = Methicillinum Sensible Staphylococcus aureus; MRSA = Methicillinum Resistant Staphylococcus aureus
